# Inhibition of glycogen phosphorylase stimulates ventromedial hypothalamic nucleus AMP‐activated protein kinase: Activity and neuronal nitric oxide synthase protein expression in male rats

**DOI:** 10.14814/phy2.13484

**Published:** 2017-12-04

**Authors:** Hussain N. Alhamami, Ayed Alshamrani, Karen P. Briski

**Affiliations:** ^1^ Department of Basic Pharmaceutical Sciences School of Pharmacy College of Health and Pharmaceutical Sciences University of Louisiana at Monroe Monroe Louisiana

**Keywords:** AMP‐activated protein kinase, glutamate decarboxylase, glycogen phosphorylase, insulin‐induced hypoglycemia, nitric oxide synthase, ventromedial hypothalamic nucleus

## Abstract

The glucose polymer glycogen is a vital fuel reserve in the brain. The mediobasal hypothalamic energy sensor AMP‐activated protein kinase (AMPK) maintains glucostasis via neurotransmitter mechanisms that suppress [*γ*‐aminobutyric acid; GABA] or stimulate [nitric oxide; steroidogenic factor‐1 (SF1)] counter‐regulatory outflow. This study investigated whether glycogen‐derived fuel supply is a critical screened variable in ventromedial hypothalamic nucleus (VMN) monitoring of neuro‐metabolic stability during glucostasis and/or insulin (I)‐induced hypoglycemia. Adult male rats were pretreated by intra‐VMN infusion of the glycogen phosphorylase inhibitor 1,4‐dideoxy‐1,4‐imino‐D‐arabinitol (DAB) before *sc* vehicle or I injection. Western blot analyses of micropunch‐dissected VMN tissue from euglycemic animals showed DAB augmentation of phosphoAMPK (pAMPK), neuronal nitric oxide synthase (nNOS), and SF‐1, but not glutamate decarboxylase_65/67_ (GAD) protein. Combinatory DAB/I treatment did not further enhance AMPK activity but significantly amplified nNOS expression relative to DAB alone. Hypoglycemic stimulation of corticosterone, but not glucagon release was prevented by DAB. Results imply that glycogen‐derived substrate fuel provision represses VMN AMPK activity and neurotransmitter signals of metabolic deficiency. Progressive augmentation of nNOS protein by DAB/I versus DAB/V intimates that “fuel‐inhibited” nitrergic neurons may exhibit increasing sensitivity to disrupted glycogen breakdown during glucoprivation versus glucostasis. nNOS and GAD reactivity to DAB/I, but not I implies that acute glycogen utilization during hypoglycemia may be sufficiently robust to avert effects on local metabolic sensory signaling. DAB/I upregulation of GAD alongside prevention of hypercorticosteronemia suggests that indicators of metabolic sufficiency may occur secondary to local compensatory adaptations to severe restriction of glucose‐derived energy.

## Introduction

In the brain, neuro‐glial energetic coupling involves cell‐type compartmentation of glucose metabolism with provision of oxidizable substrate fuel to neurons by astrocytes. Despite high energy needs, nerve cells are ironically devoid of energy stores and exhibit a truncated glycolytic pathway that favors pentose phosphate metabolism and anti‐oxidative protection over energy production (Barros 2013). The astrocyte‐neuron lactate shuttle hypothesis (ANLSH) posits that glucose, the primary energy source to the brain, is acquired from the circulation by astrocytes which either catabolize this molecule to L‐lactate for trafficking to neurons or incorporate it to the polymer glycogen (Magistretti et al. 1993; Pellerin et al. 1998). Astrocytes maintain the major glycogen depot in the mammalian brain (Phelps 1972; Koizumi 1974). Brain glycogen, although limited in mass, is the principal alternative to blood‐derived glucose as a source of energy. This energy reserve is dynamic during normal brain activity and metabolic stasis, and is an important source of lactate equivalents during states of heightened activity or glucose deficiency (Stobart and Anderson 2013).

Glucopenia poses a significant risk of neurological dysfunction and injury as a continuous glucose supply is required to maintain high‐energy nerve cell functions. Neuro‐metabolic stability is monitored in a small set of brain structures, including the mediobasal hypothalamus (MBH), where distinct populations of specialized neurons are capable of either increasing (“fuel‐inhibited”) or decreasing (“fuel‐excited”) synaptic firing in reply to reduced ambient energy substrate levels (Oomura et al. 1969; Ashford et al. 1990; Silver and Erecińska 1998). Substrate fuel screening, including lactate, in the MBH is required for optimal counter‐regulatory outflow during hypoglycemia (Borg et al. 1997, 2003). The ultra‐sensitive energy gauge 5′‐adensosine monophosphate‐activated protein kinase (AMPK) is activated by increases in the cellular AMP/ATP ratio (Kahn et al. 2005). Manipulation of MBH AMPK activity significantly alters counter‐regulatory function (Han et al. 2005; McCrimmon et al. 2008; Fan et al. 2009). The astrocyte glycogen shunt, involving sequential glucose incorporation into and liberation from glycogen prior to entry into the glycolytic pathway, is an active process accounting for a significant fraction of glucose catabolism in those cells (Walls et al. 2009; Schousboe et al. 2010). An intriguing question pertains to whether astrocyte glycogen‐derived fuel is monitored by MBH metabolic‐sensory neurons during glucostasis or hypoglycemia, particularly during the latter where depletion of the glycogen energy buffer can occur.

The inhibitory neurotransmitter *γ*‐aminobutyric acid (GABA), is reported to act within the MBH hypothalamus to diminish counter‐regulatory pancreatic glucagon and adrenomedullary catecholamine hormone responses to hypoglycemia (Chan et al. 2006, 2008; Zhu et al. 2010). Conversely, hypoglycemia‐driven counter‐regulatory outflow is enhanced by nitric oxide (NO) or steroidogenic factor‐1 (SF‐1) actions in the same locale (Fioramonti et al. 2011; Sabra‐Makke et al. 2013; Garfield et al. 2014; Routh et al. 2014). The two prominent elements of the MBH, for example, ventromedial (VMN) and arcuate (ARC) nuclei, differ markedly with respect to neurotransmitter composition, neuroanatomical connectivity within the brain, and receptivity to nutrient and endocrine signals of metabolic stability/instability. Neuroanatomical mapping of glucoprivic‐sensitive nitrergic neurons by dual Fos immunocytochemical/NADPH diaphorase histochemical staining (Dawson et al. 1991; Hope et al. 1991) showed that such cells exist in the VMN, but not ARC (Briski and Sylvester 1999). Furthermore, SF‐1 acts within the VMN to control glucose counter‐regulation (Garfield et al. 2014). The current study utilized pharmacological tools, in conjunction with high‐resolution micro‐punch dissection and high‐sensitivity western blot techniques to examine the hypothesis that diminished glycogen breakdown within the VMN elevates local AMPK activity alongside adjustments in metabolic‐sensory neuron marker protein expression. Here, adult male rats were injected into the VMN with the glycogen phosphorylase (GP) inhibitor 1,4‐dideoxy‐1,4‐imino‐D‐arabinitol (DAB) prior to subcutaneous insulin or vehicle injection. VMN tissue samples obtained from eu‐ and hypoglycemic animals were analyzed for biosynthetic enzyme [glutamate decarboxylate_65/67_ (GAD_65/67_); neuronal nitric oxide synthase (nNOS)] or neuropeptide transmitter (SF‐1) protein content.

## Methods and Materials

### Animals

Adult male Sprague Dawley rats (350–400 *g bw*) were housed in individual cages under a 14 h light/10 h dark cycle (lights on at 05.00 h), and allowed free access to standard laboratory chow diet (Harlan Teklad LM‐485; Harlan industries, Madison, WI) and tap water. All surgical and experimental protocols were conducted in accordance with NIH guidelines for care and use of laboratory animals, and approved by the ULM Institutional Animal Care and Use Committee. On day 1, each animal was implanted with a unilateral 26‐gauge stainless‐steel cannula guide (Plastics One, Inc., Roanoke, VA) aimed at the VMN [coordinates: −2.85 mm posterior to *bregma*; 0.6 mm lateral to midline; 9.0 mm ventral to skull surface], under ketamine/xylazine anesthesia (0.1 mL⁄100 *g bw*; 90 mg ketamine:10 mg xylazine⁄mL; Henry Schein Inc., Melville, NY), by motorized computer‐controlled stereotactic positioning in all three orthogonal axes, utilizing state‐of‐the‐art software (Stoelting Co., Wood Dale, IL).

### Experiment design

At 09.00 h on day 6, animals were infused into the VMN with either DAB (group 1; 150 pM (Boury‐Jamot et al. 2016); *n* = 8) or vehicle (V; 0.9% saline) (group 2; *n* = 8) in a 0.5 *μ*L total volume over 2 min (250 nL/min) via a 1.0 mm projecting internal injection cannula (Plastics One). At 09.10 h, half of the animals in each group were injected subcutaneously with neutral protamine Hagedorn insulin (I; 12.5 U/kg *bw*; Henry Schein), while the remainder were given sterile diluent (V) alone (Eli Lilly Co., Indianapolis, IN). Rats were sacrificed by decapitation at 11.10 h for trunk blood and brain collection. Each brain was snap‐frozen in liquid nitrogen‐cooled isopentane and stored at −80°C. Accuracy of cannula targeting of the VMN was verified by visual examination of consecutive frozen tissue sections cut through that structure for micropunch dissection.

### Blood glucose and hormone measurements

Whole blood glucose levels were measured with an Accuchek Advantage glucometer (Roche Diagnostic Corporation, Indianapolis, IN). Plasma glucagon and corticosterone concentrations were determined by radioimmunoassay (Briski and Nedungadi 2009).

### Western blot analyses of VMN AMPK, phosphoAMPK, and metabolic‐sensory nerve cell marker protein expression

The Palkovits micro‐punch technique (Palkovits 1985) was employed to dissect the VMN from serial 100 *μ*m‐thick sections cut through its entire rostro‐caudal length, using 0.51 mm diameter hollow needles (Stoelting, Inc., Kiel, WI). For each animal, tissue punches were collected into 20 *μ*L tissue lysis buffer (2.0% sodium dodecyl sulfate (SDS), 0.05 mol/L dithiothreitol, 10.0% glycerol, 1.0 mmol/L EDTA, 60 mmol/L Tris‐HCl, pH 7.2), and heat denatured. For each protein of interest, four separate lysate pools per treatment group were separated on 10% gradient Tris‐glycine gels (90 V, 105 min; Tris‐glycine SDS running buffer) (Magistretti et al. 1993; Matsui et al. 2012). Proteins were transblotted (30 V, overnight at 4°C; Towbin buffer) to 0.45‐*μ*m PVDF‐Plus membranes (Osmonics, Gloucester, MA). Membranes were pretreated with Western blotting signal enhancer (Pierce, Rockford, IL), blocked for 2 h with Tris‐buffer saline (TBS), pH 7.4, containing either 0.1% Tween‐20 (Sigma Aldrich, St. Louis, MO) and 2% bovine serum albumin (MP Biomedicals, Solon, OH) or 5% normal donkey serum, then incubated overnight at 4°C with primary antisera. Target proteins were probed with polyclonal antisera raised in rabbit [AMPK*α* (prod. no. 2532, 1:1000; Cell Signaling Technology, Danvers, MA); pAMPK*α* (Thr172) (prod. no. 2531, 1:1000; Cell Signal. Technol.); nNOS (prod. no. sc‐648, 1:1000; Santa Cruz Biotechnology, Inc., Santa Cruz, CA); SF‐1 (prod. no. sc‐28740; Santa Cruz Biotechnol.)] or goat [GAD 65/67 (prod. no. 7513, 1:1000; Santa Cruz Biotechnol.]. The housekeeping protein *α*‐tubulin was detected with mouse monoclonal antibodies (1:1000; EMD Millipore, Billerica, MA). Membranes were incubated for 1 h with peroxidase‐conjugated goat anti‐mouse (1:5000; PerkinElmer, Boston, MA), goat anti‐rabbit (1:5000; PerkinElmer), or donkey anti‐goat (1:5000; sc‐2020; Santa Cruz Biotechnol.) secondary antisera. After incubation with Supersignal West Femto maximum sensitivity chemiluminescent substrate (ThermoFisherScientific, Rockford, IL), signals were visualized in a Syngene G:Box Chemi (Syngene, Frederick, MD). Protein band optical densities (O.D.) were quantified in Genetool 4.01 software (Syngene) and expressed relative to *α*‐tubulin. Protein molecular weight markers were included in each Western blot analysis. Immunoblots were performed in triplicate for each protein of interest.

### Statistical analyses

Mean glucose, glucagon, corticosterone, and normalized western blot protein O.D. measures were evaluated by two‐way analysis of variance and Duncan's multiple range test. Differences in *P* < 0.05 were considered significant.

## Results

**Figure 1:**
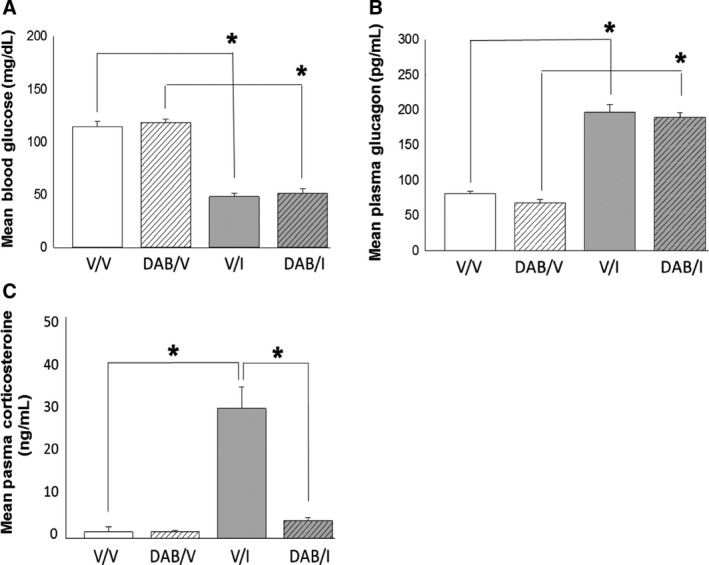
Effects of the glycogen phosphorylase inhibitor DAB administration to the VMN on circulating glucose, glucagon, and corticosterone concentrations in Eu‐ and hypoglycemic male rats. Groups of adult male rats were pretreated by DAB (150 pM) or vehicle (V) infusion into the VMN prior to subcutaneous (*sc*) injection of insulin (I; neutral protamine Hagedorn; 12.5 U/kg *bw*) or V. Bars indicate mean circulating glucose (Panel A), glucagon (Panel B), and corticosterone (Panel C) levels ± SEM for the following treatment groups (*n* = 4 rats/group): V_VMN_ plus *V*_sc_ (solid white bars); DAB_VMN_ plus *V*_sc_ (diagonal‐striped white bars); *V*_VMN_ plus *I*_sc_ (solid gray bars); DAB_VMN_ plus *I*_sc_ (diagonal‐striped gray bars). Representative target protein and *α*‐tubulin blots are shown with each graph. *P < 0.05. VMN, Ventromedial Hypothalamic Nucleus; DAB, 4‐Dideoxy‐1,4‐Imino‐D‐Arabinitol.

Figure 1 illustrates effects of infusion of the GP inhibitor DAB to the VMN on blood glucose (Panel 1A) and plasma glucagon (Panel 1B) and corticosterone levels (Panel 1C) in vehicle (V)‐ and insulin (I)‐injected male rats. Results indicate that DAB did not alter circulating glucose, glucagon, or corticosterone levels in euglycemic animals [DAB/V versus V/V]. Outcomes show that I injection decreased glucose concentrations, while elevating counter‐regulatory hormone secretion. Although the magnitude of glucose and glucagon responses to I dosing did not differ between DAB/I versus V/I groups, DAB normalized corticosterone secretion in hypoglycemic animals.

**Figure 2:**
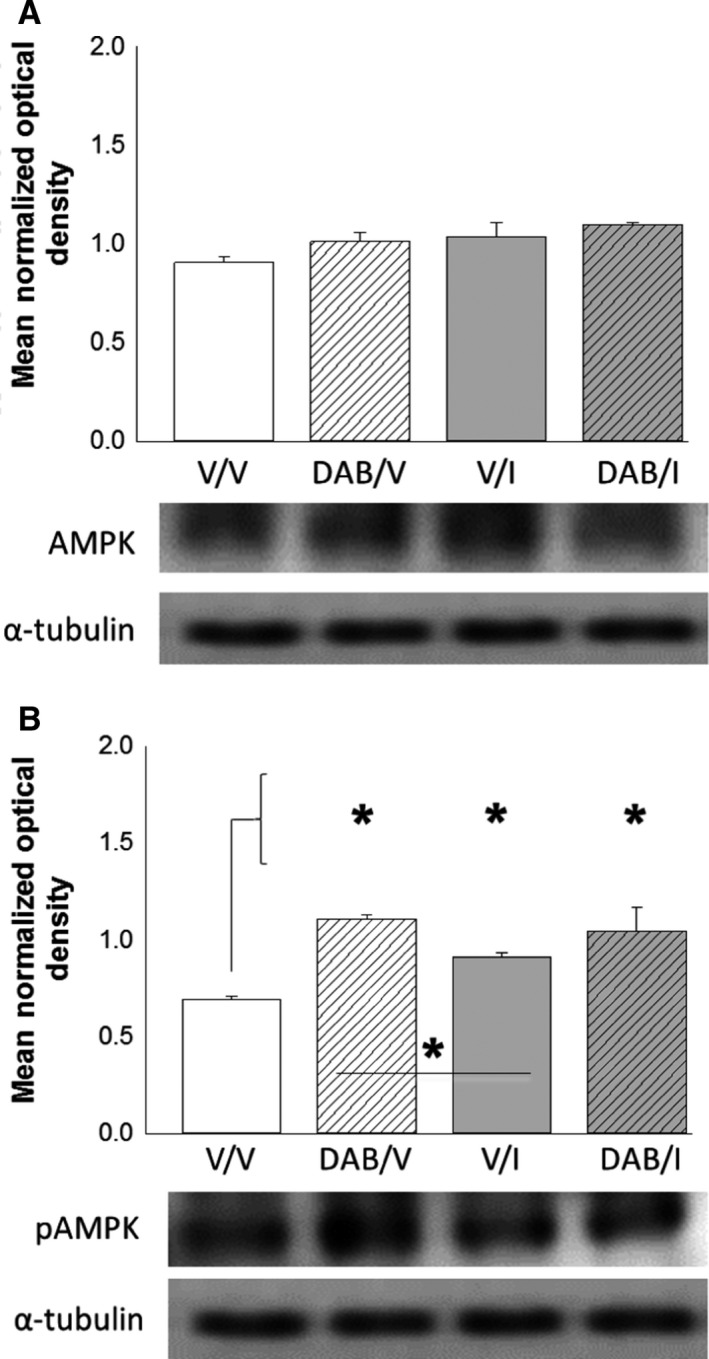
Effects of DAB on VMN AMPK activity in Eu‐ and hypoglycemic male rats. Groups of adult male rats were pretreated by DAB or V infusion into the VMN prior to *sc* injection of I (12.5 U/kg *bw*) or V. The VMN was collected by micro‐punch dissection from serial 100 micron‐thick frozen sections for Western blot analyses of AMPK (Panel A) and phosphoAMPK (pAMPK; Panel B). Bars depict illustrate mean normalized protein O.D. measures ± SEM for groups of animals (*n* = 4 rats/group) treated as follows: *V*_VMN_ plus *V*_sc_ (solid white bars); DAB_VMN_ plus *V*_sc_ (diagonal‐striped white bars); *V*_VMN_ plus *I*_sc_ (solid gray bars); DAB_VMN_ plus *I*_sc_ (diagonal‐striped gray bars). Representative target protein and *α*‐tubulin blots are shown with each graph. * *P* < 0.05. VMN, Ventromedial Hypothalamic Nucleus; DAB, 4‐Dideoxy‐1,4‐Imino‐D‐Arabinitol. AMPK, 5′‐adenosine ‐monophosphate‐activated protein kinase**.**

**Figure 3:**
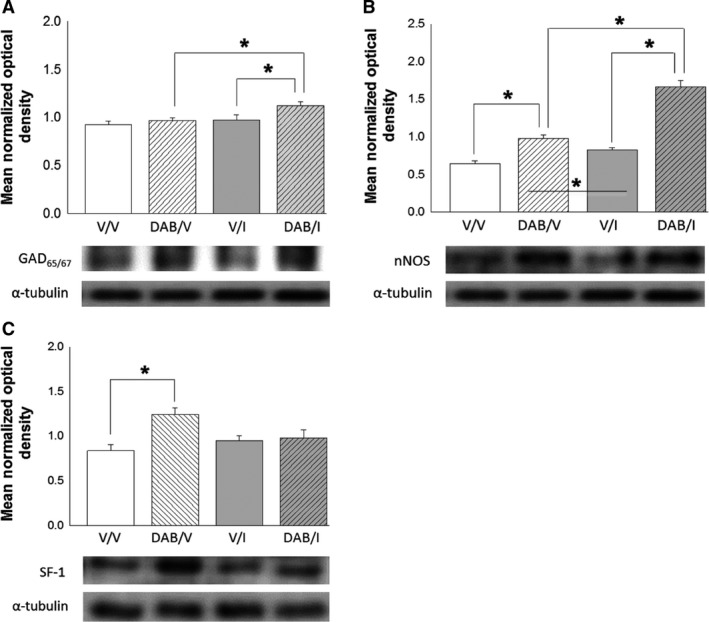
Effects of DAB on VMN GAD_65/67_, nNOS, and SF‐1. Protein expression in Eu‐ and hypoglycemic male rats. Microdissected VMN tissue from groups of male rats pretreated by intra‐VMN DAB or V infusion prior to *sc* injection with I or V were analyzed by Western blot for GAD_65/67_ (Panel A), nNOS (Panel B), and SF‐1 (Panel C) protein content. Bars depict illustrate mean normalized protein O.D. measures ± SEM. for the following treatment groups (*n* = 4 rats/group): *V*_VMN_ plus *V*_sc_ (solid white bars); DAB_VMN_ plus *V*_sc_ (diagonal‐striped white bars); *V*_VMN_ plus *I*_sc_ (solid gray bars); DAB_VMN_ plus I_sc_ (diagonal‐striped gray bars). Representative target protein and *α*‐tubulin blots are depicted below each graph. *P < 0.05. DAB, 1,4‐dideoxy‐1,4‐imino‐D‐arabinitol; VMN, ventromedial hypothalamic nucleus; GAD_65/67_, glutamate decarboxylase_65/67_; nNOS; neuronal nitric oxide synthase.

As shown in Figure 2, intra‐VMN delivery of DAB did not modify VMN AMPK protein content in vehicle‐ or I‐injected rats (Panel 2A), but significantly augmented local pAMPK profiles in both eu‐ and hypoglycemic animals. I injection alone increased pAMPK expression in this site, albeit to a lesser extent compared to DAB. Data in Figure 3 depict effects of DAB on VMN GAD_65/67_ (Panel 3A), nNOS (Panel 3B), and SF‐1 (Panel 3C) protein expression. Outcomes show that DAB increased nNOS and SF‐1 levels in the VMN compared to vehicle‐pretreated animals, whereas GAD_65/67_ levels were refractory to treatment with this GP inhibitor [DAB/V versus V/V]. I‐injected rats showed no change in GAD_65/67_, nNOS, or SF‐1 protein profiles compared to V/V animals. DAB‐pretreated hypoglycemic rats exhibited augmented VMN accumulation of GAD_65/67_ and nNOS proteins relative to groups treated with either DAB [DAB/I vs. DAB/V] or I [DAB/I vs. V/I] alone.

## Discussion

Vital nerve cell high energy‐consuming activities are sustained by astrocyte trafficking of the oxidizable end‐product of astrocyte glycolysis, l‐lactate, for mitochondrial respiration. The astrocyte glycogen depot is the primary alternative fuel to blood‐derived glucose; this reserve is actively remodeled during normal brain activity and metabolic stability (Swanson et al. 1989) and provides a critical supply of lactate equivalents during heightened brain activity or glucose deficiency (Gruetter 2003; Brown 2004; Brown and Ransom 2007). MBH screening of lactate availability shapes counter‐regulatory responses to hypoglycemia (Borg et al. 2003). Here, we examined the premise that glycogen‐derived fuel accessibility regulates VMN AMPK activity and metabolic effector neurotransmitter signaling during glucose abundance and/or deficiency. Results show that VMN pAMPK expression was elevated by either intra‐VMN DAB or subcutaneous I administration, but was refractory to additive treatment effects. VMN SF‐1 and nNOS proteins were significantly enhanced by DAB alone; DAB/I further augmented the latter protein profile. VMN GAD_65/67_ levels were higher in DAB/I versus V/I or DAB/V groups. Results show that during energy homeostasis, dynamic glycogen turnover represses VMN AMPK activity and neurotransmitter signals of metabolic deficiency. Intensified DAB‐induced stimulation of nNOS expression in the presence versus absence of hypoglycemia implies that “fuel‐inhibited” nitrergic neurons exhibit amplified sensitivity to glycogen depletion during this metabolic stress. Reactivity of VMN NOS and GAD_65/67_ proteins to DAB/I but not hypoglycemia alone suggests that glycogen mobilization may be sufficient over an acute time frame to preserve normal nitrergic and GABAergic signaling. DAB/I upregulation of GAD alongside prevention of hypercorticosteronemia may likely reflect local compensatory adaptations to severity of concurrent restriction of blood and glycogen energy sources.

Current data show that DAB did not modify VMN total AMPK protein, but significantly upregulated pAMPK expression to an extent that exceeded levels measured in hypoglycemic animals. VMN pAMPK content was not different between groups treated with DAB/I versus DAB alone, suggesting that capacity for further enzyme phosphorylation may be limited. Alternatively, inhibition of glycogen breakdown alongside hypoglycemia may prompt compensatory metabolic adjustments, such as decreased energy expenditure, etc., which prevent further divergence between cellular AMP and ATP levels and thus curb further amplification of sensor activity. In the absence of experimental confirmation of the efficacy of the current DAB treatment regimen in inhibiting VMN GP enzyme activity, alternative explanations for observed drug effects on target protein expression, including off‐target actions, should not be discounted at this time. Likewise, the inability to measure and compare VMN glycogen content between vehicle‐ and DAB‐treated animals is a limitation of this study.

DAB‐mediated enhancement of VMN gluco‐regulatory neurotransmitter signaling in euglycemic rats affirms that local “fuel‐inhibited” metabolic‐sensory neurons are alert to reductions in astrocyte‐generated energy substrate stream. As astrocytes shuttle a sizable fraction of internalized glucose through the glycogen shunt ahead of catabolism to lactate, it is plausible that DAB‐inhibited glycogen breakdown decreases net glucose entry into the glycolytic pathway, thereby diminishing trafficable lactate. In this scenario, DAB‐mediated VMN nitrergic and SF‐1 neuron activation could be reasonably construed to reflect reduced lactate uptake. NO facilitates optimal reactivity of MBH “fuel‐inhibited” neurons to diminished glucose (Fioramonti et al. 2010, 2011), but evidence that nitrergic neurons express the requisite molecular machinery (e.g., AMPK, etc.) to perform that monitoring function remains elusive. VMN SF‐1 neurons function downstream of parabrachial nucleus metabolic‐sensory cholecystokinin neurons to control counter‐regulation (Garfield et al. 2014); outcomes here imply that these cells may also likely react to local signals of neuro‐glial metabolic instability. On the other hand, Walls et al. (2009) report that DAB elicits compensatory augmentation of glycolytic activity in vitro, thereby promoting relatively higher lactate yield despite glycogen shunt blockade. Because we did not measure DAB effects on net lactate transport between VMH astrocyte and nerve cell compartments here, we cannot set aside the possibility that astrocytes may communicate attenuated glycogen disassembly to “fuel‐inhibited” neurons by signals other than or in addition to trafficked lactate volume, such glio‐transmitter signaling. For example, octadecaneuropeptide (ODN), a cleavage product of the astrocyte transmitter diazepam‐binding inhibitor (DBI), acts on hypothalamic substrates to diminish food intake and blood glucose levels (Lanfray et al. 2013). At present, it is unclear if VMN astrocytes release DBI to signal changes in glycogen breakdown rates or net mass.

Brain glycogen depletion can occur under a variety of circumstances including hypoglycemia, ischemia, exhaustive exercise, body temperature extremes, prolonged wakefulness, or amphetamine exposure (Hutchins and Rogers 1970; Swanson et al. 1989; Kong et al. 2002; Canada et al. 2011; Fioramonti et al. 2011; Matsui et al. 2012). Astrocyte communication of actual or impending glycogen exhaustion is arguably neuro‐protective, as such signals would likely trigger local or brain‐wide cellular adaptations such as reduction/partitioning of energy expenditure and/or reliance upon alternative fuels, alongside systemic mechanisms (e.g., glucose counter‐regulation) designed to replenish this energy reserve. Further studies are needed to address whether VMN NOS and/or SF‐1 neurons may respond exclusively to hypoglycemia‐associated glycogen exhaustion or are activated by depletion of this reserve regardless of initiating circumstances. It would also be informative to learn if, aside from their function within pathways that regulate body‐wide corrective functions, these neurotransmitters act on local substrates to optimize cellular acclimation to diminished glycogen‐derived substrate fuel.

Present data show that insulin‐induced hypoglycemia (IIH) did not alter GAD_65/67_, nNOS, or SF‐1 protein expression in the male rat VMN. Available reports disclose that putative “fuel‐excited” GABAergic neurons react to exogenous glucose infusion to the MBH, as indicated by increased extracellular GABA and circulating glucose levels alongside diminished counter‐regulatory outflow (Walls et al. 2009). Moreover, hyperinsulemic‐hypoglycemic clamping effectively reduces MBH tissue GABA concentrations (Chan et al. 2008). Those studies differ from the current work in that they involved analysis of a more expansive tissue area, for example, MBH versus VMN; discrepant outcomes thus point to the possibility of dissimilar VMN versus ARC GABAergic nerve cell reactivity to hypoglycemia. There remains a need to determine if and where within the MBH GABA neurons express AMPK and/or other metabolo‐sensory molecular markers. Alternatively, hypoglycemia may lower VMN GABA levels in the absence of a measurable impact on GAD_65/67_ expression. However, we observed that VMN nNOS protein levels were refractory to hypoglycemia, others reported augmenting effects of hypoglycemia on MBH enzyme activity and tissue NOS levels (Fioramonti et al. 2011). Differential outcomes could reflect, in part, insulin formulation/dosage, time interval between insulin injection and tissue procurement, and/or method/specificity of analysis, for example, net enzyme biochemical activity versus Western blot measurement of tissue neuron‐specific enzyme protein. Further studies are needed to address whether hindbrain parabrachial nucleus input is required for reactivity of VMN SF‐1 neurons to hypoglycemia. It is insightful to note dissimilar reactivity of VMN nNOS and SF‐1 proteins to intra‐VMN DAB treatment amid eu‐ versus hypoglycemia. A probable explanation is that the extent of inhibition of glycogen breakdown achieved by this pharmacological paradigm may exceed that occurring over a two hour period after insulin injection owing to reduced glycogen mass. It is possible that one or both proteins may be upregulated later as hypoglycemia persists and glycogen is progressively spent. Evidence that nNOS expression is further enhanced to a substantial degree by combinatory DAB plus I versus DAB alone is consistent with that view. Additional work is warranted to assess whether VMN nitrergic neurons show discriminative reactivity to magnitude of decline in either glycogen‐derived glycosyl units or net glucose (acquired from blood plus glycogen) molecules provided to the astrocyte glycolytic pathway. It is interesting to note that VMN GAD_65/67_ protein levels were elevated in response to DAB plus insulin. This unpredicted result could reflect paracrine communication from hyper‐activated “fuel‐inhibited” neurons to these cells, resulting in activation of the latter as a physiological “brake”, or alternatively, GABA nerve cell signaling of positive cellular energetic acclimation to this metabolic stress.

Data show that intra‐VMN DAB administration to eu‐ or hypoglycemia animals did not modify circulating glucose and glucagon concentrations, despite alterations in gluco‐regulatory neurotransmitter enzyme/neuropeptide expression. These findings are consistent with earlier reports that administration of the AMPK activator 5‐aminoimidazole‐4‐carboxamide ribonucleotide (AICAR) to the MBH did not elevate counter‐regulatory hormone secretion (McCrimmon et al. 2004). On the other hand, more far‐reaching delivery of AICAR to the brain via the lateral cerebral ventricle effectively upregulated glucagon output (Han et al. 2005). It is thus conceivable that metabolic deficit stimuli of MBH origin are insufficient to stimulate release of this counter‐regulatory hormone in the absence of coincident signals from additional neural loci. Interestingly, DAB/I treatment was observed to reverse hypoglycemic stimulation of corticosterone release, indicating inhibition of the hypothalamic‐pituitary‐adrenal axis by sequelae of decreased VMN glycogen breakdown, whereas DAB alone had no impact on this hormone profile. We did not predict this very intriguing outcome, and aim to identify underlying mechanisms in ongoing research. At present, we theorize that this normalization may be causally associated with upregulation of local GAD_65/67_ expression owing to local metabolic/energetic adaptations to concurrent diminishment of blood‐ and glycogen‐derived glucose for production and trafficking of L‐lactate.

In summary, this research offers novel proof that glycogen‐derived fuel is a key variable in VMN screening of brain neuro‐metabolic stability during glucose plenty and shortage. Data show that pharmacological interruption of local glycogen turnover stimulates VMN AMPK activation and expression of “fuel‐inhibited” metabolic‐sensory neuron marker proteins in euglycemic animals, suggesting that glycogen‐derived fuel supply curbs local neurotransmitter signals of metabolic deficiency. Evidence for amplification of nNOS protein profiles by DAB plus insulin versus DAB alone implies that nitrergic neurons exhibit escalating sensitivity to increasing diminution of glycogen breakdown. Refractoriness of nNOS and GAD_65/67_ proteins to hypoglycemia alone supports the view that glycogen mobilization over an initial 2 h period after insulin injection may be sufficiently robust to have negligible impact on GABA‐ or nitrergic signaling.

## Conflict of Interest

The authors have no conflicts of interest to report.
